# Supplementary material for: Donor-Derived Cell-Free DNA as a Non-Invasive Biomarker for Graft Rejection in Kidney Transplant Recipients: A Prospective Study among the Indian Population
Source: Diagnostics (Basel). 2023 Nov 27;13(23):3540. doi: 10.3390/diagnostics13233540 (PMC10706139; doi:10.3390/diagnostics13233540)
Supplement: Supplementary file 1 [file diagnostics-13-03540-s001.zip › diagnostics-2604789-supplementary.pdf]

Donor-Derived Cell-Free DNA as a Non-Invasive Biomarker for Graft Rejection in Kidney Transplant Recipients: A Prospective Study among the Indian Population

Supplementary Table S1: Primers and Probes sequences of the selected assays

**Assay Name**	**SNPs**	**Forward Primer**	**Reverse Primer**	**Probe A (5`-FAM/3`-BHQ1)**	**Probe B (5`-HEX/3`-BHQ1)**	**$MgCl_2$***
**S82**	**rs10228737**	Tttgcacttgacgcaccagc	ccgaggcagaggaaggaagtg	tgcAatgagagcagaggcct	catCgcagccctcctgca	No
**S87**	**rs10734083**	Ggcatctgaattcaagctttggtc	ttcttctagttggtctggtaggct	aggcttgtacactCtccccc	acactgggatgggggaAagt	Yes
**S99**	**rs12064796**	Ggcaaagtgggcaagggtct	gcctcctaaagcttgagccaca	ttggggccaGgtacctgg	tggggccaAgtacctggt	No
**S103**	**rs4632826**	Agctttcttgcttctgcccca	gggtgccattgcccagagat	ccctggggccatcaGgtt	ccctggggccatcaAgttt	Yes
**S108**	**rs11610836**	Acactcctgctgcgtgtctg	ttcctccccaccactcccat	ggtcccagctggtCgtgg	atgctccccacAaccagct	Yes

Note: Capital letters in probe sequences indicate the position of inferred SNP. * Requirement of additional $MgCl_2$ in the reaction.
